# A case–control study on the association between adherence to a Mediterranean-style diet and breast cancer

**DOI:** 10.3389/fnut.2023.1140014

**Published:** 2023-07-18

**Authors:** Omid Sadeghi, Niloofar Eshaghian, Sanaz Benisi-Kohansal, Leila Azadbakht, Ahmad Esmaillzadeh

**Affiliations:** ^1^Nutrition and Food Security Research Center, Department of Community Nutrition, School of Nutrition and Food Science, Isfahan University of Medical Sciences, Isfahan, Iran; ^2^Student Research Committee, Isfahan University of Medical Sciences, Isfahan, Iran; ^3^Department of Community Nutrition, School of Nutritional Sciences and Dietetics, Tehran University of Medical Sciences, Tehran, Iran; ^4^Obesity and Eating Habits Research Center, Endocrinology and Metabolism Molecular-Cellular Sciences Institute, Tehran University of Medical Sciences, Tehran, Iran

**Keywords:** Mediterranean diet, breast cancer, case–control study, food frequency questionnaire, diet

## Abstract

**Background:**

Previous studies on the association between diet and breast cancer are mostly from Western populations, and data from Middle East countries are scarce, where the prevalence of breast cancer is high; therefore, it ranks first among other cancers. This population-based case–control study aimed to investigate the relationship between a Mediterranean-style diet and breast cancer among Iranian women.

**Methods:**

In the current study, 350 new cases of breast cancer and 700 age- and socioeconomic status-matched controls were enrolled. We evaluated the dietary intakes of participants by using a 106-item Willett-format semi-quantitative dish-based food frequency questionnaire (SQ-FFQ). We calculated the Mediterranean diet score according to the dietary intakes of participants. In addition, using pre-tested questionnaires, we collected information on potential confounding variables.

**Results:**

In this study, we found a significant inverse association between the Mediterranean diet and breast cancer so that after controlling for potential confounders, individuals in the highest tertile of the Mediterranean diet score compared with those in the lowest tertile were 57% less likely to have breast cancer [odds ratio (OR): 0.43, 95% confidence interval (CI): 0.28–0.67]. Such an inverse association was also observed for postmenopausal women. Similarly, after controlling for potential confounding variables, high adherence to the Mediterranean dietary pattern was associated with lower odds of breast cancer (OR: 0.37, 95% CI: 0.23–0.60). However, this relationship was not significant among premenopausal women.

**Conclusion:**

We found that adherence to Mediterranean dietary pattern was associated with reduced odds of breast cancer. Studies with prospective design are needed to further examine this association.

## Introduction

Mediterranean dietary pattern is one of the healthy dietary patterns which is associated with high intakes of plant foods, olive oil, fish, and sea foods, low-to-moderate consumption of milk and dairy products, low consumption of poultry, red and processed meat, and moderate consumption of wine (during meals) (1, 2). This dietary pattern is rich in fiber, antioxidants, omega-3 polyunsaturated fatty acids (PUFAs), vitamins B and E, and minerals such as magnesium (3, 4). Previous studies have shown that these nutrients have beneficial effects on chronic diseases such as cardiovascular diseases (CVDs), hypertension, and some cancers (5–7). For instance, in the UK Women's Cohort Study, the Mediterranean dietary pattern was inversely associated with colorectal cancer risk (8). In addition, it has been shown that the Mediterranean diet has protective effects against prostate, gastric, and pancreatic cancers (9–11).

Given the anti-inflammatory effects of the Mediterranean diet, it may have a benefit for the prevention of cancers such as breast cancer. However, the Mediterranean dietary pattern is rich in phytoestrogens that may adversely affect breast cancer incidence (12). In addition, the findings from previous studies on the relationship between adherence to the Mediterranean dietary pattern and the risk of breast cancer are conflicting. In a systematic review in 2015, Farsinejad et al. reported that there is no sufficient data to reach a definite conclusion about the effect of the Mediterranean diet on the risk of breast cancer in premenopausal and postmenopausal women (13). However, in the PREDIMED trial, it has been shown that adherence to the Mediterranean dietary pattern combined with extra-virgin olive oil has beneficial effects on the prevention of breast cancer (14). In addition, a case–control study reported an inverse association between the Mediterranean diet and breast cancer (15). However, in a prospective cohort study in Sweden, no significant association between the Mediterranean dietary pattern and the risk of breast cancer was reported (16). In addition to discrepant findings, most studies on the association between the Mediterranean diet and the risk of breast cancer have been conducted in Western countries, whereas no study is available on the Middle Eastern population where the incidence of breast cancer is estimated to be high. In Iran, the prevalence of breast cancer is high, and its incidence rate is 35.8 per 100,000 population (17). In Mediterranean countries, adherence to the Mediterranean dietary pattern is common; however, adherence to a Mediterranean-style diet may have beneficial effects on the Middle Eastern population. Therefore, our study aimed to investigate the relationship between adherence to the Mediterranean dietary pattern and breast cancer in Iranian women.

## Materials and methods

### Study population

We conducted a population-based case–control study on women older than 30 years living in Isfahan, Iran. In this study, breast cancer cases were diagnosed by mammography findings and physical examination within the last 6 months at most. From July 2013 to July 2015, cases of breast cancer were recruited from patients who were referred to private clinics or hospitals in Isfahan, Iran. By considering that an unhealthy diet might enhance the risk of breast cancer by 1.5 times and the type I error of 5%, the study power of 80%, the common ratio of 0.25 and 2 for the ratio of controls to cases, we required 350 cases of breast cancer and 700 healthy controls for the current study. Cases were undergoing surgical resection of breast tumors, chemotherapy, radiotherapy, or all of them. In the current study, cases were those who had a primary breast tumor with aggressive behavior, and its histology was available and accessible in patients' medical records. We did not include patients who had a history of neoplastic lesions or cysts (except current breast cancer), and those patients who had a history of hormone replacement therapy. In addition, patients who had a special diet were not included. Using the cluster method sampling, age (±5 year) adjusted controls were randomly selected from healthy women who had no relationship with breast cancer patients and had no family history of breast cancer. In addition to age, we did our best to match controls in terms of their place of residence as a proxy measure of socioeconomic status (SES) with the cases. In this study, controls were Iranian women who had no history of malignancy, medical disorder, cysts, and hormone replacement therapy and who did not have a special diet from the general adult population. Finally, 350 cases and 700 controls were included in this study. An informed consent form was provided to participants, and they were asked to sign it. The Ethical Committee of Isfahan University of Medical Sciences, Isfahan, Iran has ethically approved the present study.

### Assessment of dietary intakes

In this study, the dietary intakes of participants were collected by using a 106-item Willett-format semi-quantitative dish-based food frequency questionnaire (SQ-FFQ). This questionnaire has been specifically designed and validated for the Iranian population (18). Information on the design and validity of this questionnaire has been reported elsewhere (19). In the current study, a trained nutritionist completed the questionnaires through a face-to-face interview. Participants reported their dietary intake of foods and mixed dishes according to 9 multi-choice frequency response categories ranging from “never or less than once per month” to “≥ 12 times per day.” For the food list, categories of frequency response ranged from 6 to 9 choices. For low-consumption foods, we eliminated the high-frequency categories, while for high-consumption foods, the number of multiple-choice categories was increased. Finally, for all food items, we calculated daily intakes and then using household measures converted them to grams per day (20). We computed daily values for all food items according to the composition of foods, average frequency of food consumption, and portion size of foods. We computed nutrient intake by summing up the nutrients of all foods. In this study, we obtained nutrient intake by using Nutritionist IV software, which was modified for Iranian foods.

### Construction of the Mediterranean diet score

Based on the methodology by Trichopoulou (21, 22), we computed scores of the Mediterranean diet based on nine components [positive components: fish, fruits, vegetables, legumes, nuts, and ratio of monounsaturated fatty acids (MUFAs) to saturated fatty acids (SFAs) and negative components: grains, dairy, and meats (poultry, red meat, and processed meat)]. If participants were at the top median intakes of positive components and bottom median intakes of negative components, they received a score of 1. Participants received a score of 0 if they were at the top median intakes of negative components and bottom median intakes of positive components. By summing up the score of each component, we obtained the total score for the Mediterranean diet.

### Assessment of breast cancer

All breast cancer patients were Iranian women who were recently diagnosed with breast cancer (stages I–IV). For all of them, the local or invasive status of breast cancer was determined by using mammography and physical examination. Mammography is an imaging method using X-ray that is used to diagnose diseases related to the breast (23). The adverse side effects of exposing breasts to radiation with this method are very low and can be ignored. To perform mammography, patients were placed in standing, vertical, and horizontal situations. For a few seconds, the breast was pressed between pages, and then, the photograph was taken.

### Evaluation of other variables

By using a pre-tested questionnaire, we collected information about participants' age, education, marital status, menopausal status, region, family history of breast cancer, smoking, consumption of alcohol, and disease history. This questionnaire was completed for all participants through face-to-face interviews. Regarding anthropometric assessments, participants' weight was measured using a digital scale to the nearest 100 grams. Weight measurements were taken while the participants were minimally clothed and without shoes. By using a tape meter that was mounted on the wall, we measured the height of participants to the nearest 0.5 cm. Height measurement was performed while the participants were standing normally without shoes. In addition, we calculated body mass index (BMI) according to the following formula: “weight (kilograms)/height (meters squared).” In the current study, a short form of the International Physical Activity Questionnaire (IPAQ) was used to evaluate the physical activity level of the participants. Participants were classified as physically inactive (having <1 h of moderate physical activity per week) or physically active (having 1 h or more of moderate physical activity per week).

### Statistical analysis

At first, by using an independent sample *t*-test and chi-square test, cases and controls were compared in terms of general characteristics. Then, participants were classified according to tertile cutoff points of the Mediterranean diet score. To investigate the differences of continuous variables across tertiles of the Mediterranean diet score, we used one-way ANOVA. In addition, to evaluate categorical variable distribution across tertiles of the Mediterranean diet score, the chi-square test was employed. To determine the relationship between adherence to the Mediterranean diet and breast cancer, we used binary logistic regression in adjusted models. Age and energy intake were considered in the first model. In the second model, we made further adjustments for participants' education (non-educated/educated), marital status (single/married), menopausal status (premenopausal/postmenopausal), region (rural/urban), family history of breast cancer (no/yes), smoking (non-smoker/smoker), consumption of alcohol (no/yes), physical activity, socioeconomic scores (continuous), and disease history (no/yes). In addition, the final model was adjusted for BMI, to remove the confounding effect of obesity from the relationship between the Mediterranean diet and breast cancer. In all analyses, the reference group was those who were in the first tertile of the Mediterranean diet score. In the current study, to obtain the overall trend of odds ratios of breast cancer across tertiles of the Mediterranean diet score, we considered these categories as an ordinal variable. In addition, the analysis of logistic regression was stratified according to menopausal status (premenopausal/postmenopausal). By using SPSS software (version 18), we performed all statistical analyses. In this study, the significance level was *P*-value < 0.05.

## Results

Table 1 presents the general characteristics of cases and controls. Compared with the control group, cases are more likely to be older, single, postmenopausal, smoker, non-educated, and have a lower BMI and family history of breast cancer. We found no other significant differences between cases and controls.

**Table 1:** General characteristics of cases and controls.

**Variables**	**Cases (*n* = 350)**	**Controls (*n* = 700)**	*P*-value^*^
Age (year)	65.2 ± 11.2	61.0 ± 10.3	<0.001
Social economic status	12.8 ± 5.2	13.4 ± 5.5	0.09
BMI (kg/m^2^)	21.8 ± 4.8	25.5 ± 5.0	<0.001
Region (urban) (%)	36.0	36.1	0.96
Marital status (single) (%)	25.1	11.3	<0.001
Education (educated) (%)	17.4	28.9	<0.001
Disease history (yes) (%)	10.3	8.7	0.40
Physical activity (inactive) (%)	31.7	34.4	0.38
Family history of breast cancer (yes) (%)	9.4	3.4	<0.001
Menopausal status (postmenopausal) (%)	88.3	77.4	<0.001
Smoking (smoker) (%)	17.4	13.0	0.05
Alcohol (alcoholic) (%)	4.6	7.4	0.07

Data are presented as mean (SD) or percent.

BMI, body mass index.

* Obtained from independent sample t-test or chi-square, where appropriate.

Table 2 shows the general characteristics of participants across tertiles of the Mediterranean diet score. Women with the highest tertile of the Mediterranean diet score were more likely to be younger, married, educated, alcohol consumers, living in urban areas, having a higher BMI and socioeconomic score and were less likely to be postmenopausal and have a history of disease compared with those in the lowest category. There was no other significant difference in this regard.

**Table 2:** General characteristics of the study participants across tertiles (T) of Mediterranean diet scores.

	**T_1_**	**T_2_**	**T_3_**	*P* ^*^
Age (year)	65.31 ± 10.06	61.78 ± 11.01	60.66 ± 10.80	<0.001
BMI (kg/m^2^)	23.25 ± 5.27	24.49 ± 5.44	25.14 ± 4.90	<0.001
Socioeconomic score	11.41 ± 4.48	12.76 ± 4.94	15.72 ± 6.16	<0.001
Region (urban) (%)	24.7	33.7	51.0	<0.001
Marital Status (single) (%)	21.6	15.4	11.0	0.009
Education (Educated) (%)	16.6	19.8	41.3	<0.001
Disease history (yes) (%)	12.2	9.5	6.0	0.03
Physical activity (inactive) (%)	37.5	32.6	31.0	0.20
Family history of breast cancer (yes) (%)	7.8	5.3	3.3	0.06
Menopausal status (post) (%)	88.5	79.3	76.3	<0.001
Smoking (smoker) (%)	17.2	13.9	12.7	0.25
Alcohol consumption (yes) (%)	1.4	5.7	12.7	<0.001

Data are presented as mean (SD) or percent.

BMI, body mass index.

* Obtained from one-way analysis of variance (ANOVA) or chi-square, where appropriate.

Table 3 indicates the dietary intakes of women across tertiles of the Mediterranean diet score. Compared with women in the bottom tertile of the Mediterranean diet score, those in the top tertile had higher intakes of fish, legumes, nuts, fruits, vegetables, MUFAs, PUFAs, and fiber and lower intakes of grains, dairy products, and SFAs. In addition, the ratio of MUFAs-to-SFAs was different across tertiles of the Mediterranean diet score. We found no other significant differences in this regard.

**Table 3:** Dietary intakes of study participants across tertiles (T) of Mediterranean diet scores.

	**T_1_**	**T_2_**	**T_3_**	*P* ^*^
**Food groups (g/d)**				
Fruits	131.4 ± 161.1	150.6 ± 135.5	219.2 ± 160.3	<0.001
Vegetables	44.6 ± 38.4	76.1 ± 59.0	124.0 ± 92.8	<0.001
Legume	8.9 ± 8.9	13.8 ± 11.2	21.9 ± 20.8	<0.001
Nuts	0.3 ± 1.0	2.0 ± 4.7	4.7 ± 8.2	<0.001
Fish	3.9 ± 11.2	10.6 ± 44.7	12.4 ± 16.0	0.002
Grains	441.2 ± 155.2	440.5 ± 163.0	409.0 ± 148.6	0.01
Dairy products	251.0 ± 186.1	211.5 ± 138.2	242.5 ± 143.0	0.001
Meat	89.3 ± 73.3	89.8 ± 87.7	88.8 ± 66.2	0.98
**Nutrients**				
Energy (kcal/d)	2,301 ± 715	2,286 ± 712	2,265 ± 637	0.81
Protein (g/d)	77.7 ± 27.2	76.8 ± 30.3	78.3 ± 27.3	0.77
Carbohydrate (g/d)	314.4 ± 112.2	318.6 ± 110.9	318.0 ± 97.0	0.86
Fat (g/d)	87.4 ± 35.3	84.3 ± 36.9	82.1 ± 31.1	0.17
SFA (g/d)	52.7 ± 26.4	48.0 ± 29.3	41.4 ± 19.5	<0.001
MUFA (g/d)	19.4 ± 7.7	20.0 ± 8.2	21.5 ± 8.8	0.004
PUFA (g/d)	8.4 ± 4.2	9.5 ± 5.0	12.4 ± 10.6	<0.001
MUFA/SFA	0.4 ± 0.1	0.5 ± 0.2	0.6 ± 0.3	<0.001
Fiber (g/d)	20.6 ± 7.4	22.2 ± 7.9	24.2 ± 7.9	<0.001

Data are presented as mean (SE).

SFA, saturated fatty acid; MUFA, monounsaturated fatty acid; PUFA, polyunsaturated fatty acid; g/d, gram/day; kcal/d, kcal/day.

* Obtained from one-way ANOVA.

Table 4 illustrates multivariable-adjusted odds ratios (ORs) and 95% confidence intervals (CIs) for breast cancer across tertiles of the Mediterranean diet score. We found a significant inverse association between the Mediterranean diet and breast cancer (OR: 0.34, 95% CI: 0.23–0.48). Such association was also seen after adjusting for demographic variables, menopausal status, energy intake, physical activity, and BMI; such that women in the top tertile of the Mediterranean diet score compared with those in the bottom tertile had 57% lower odds of breast cancer (OR: 0.43, 95% CI: 0.28–0.67). Such association was also observed for postmenopausal women; such that after controlling for potential confounding variables, postmenopausal women in the highest tertile of the Mediterranean diet score compared with those in the lowest tertile were 63% less likely to have breast cancer (OR: 0.37, 95% CI: 0.23–0.60). However, among premenopausal women, we found no significant association in this regard.

**Table 4:** Multivariable-adjusted odds ratios (ORs) and 95% confidence intervals (CI) for breast cancer across tertiles (T) of Mediterranean diet scores.

	**Mediterranean diet scores**		
	**T**_1_	**T**_2_	**T**_3_
**Total**			
Crude	1	0.65 (0.48–0.88)	0.34 (0.23–0.48)
Model 1	1	0.73 (0.53–1.01)	0.38 (0.26–0.56)
Model 2	1	0.74 (0.53–1.03)	0.42 (0.28–0.63)
Model 3	1	0.78 (0.54–1.12)	0.43 (0.28–0.67)
**Premenopausal women**			
Crude	1	0.68 (0.29–1.58)	0.51 (0.22–1.17)
Model 1	1	0.76 (0.31–1.83)	0.61 (0.25–1.44)
Model 2	1	0.88 (0.33–2.35)	0.76 (0.28–2.06)
Model 3	1	1.45 (0.43–4.83)	1.94 (0.57–6.54)
**Postmenopausal women**			
Crude	1	0.64 (0.46–0.89)	0.33 (0.22–0.49)
Model 1	1	0.69 (0.49–0.98)	0.35 (0.23–0.52)
Model 2	1	0.70 (0.49–1.00)	0.37 (0.24–0.58)
Model 3	1	0.73 (0.50–1.07)	0.37 (0.23–0.60)

Data are presented as OR and 95% CI.

Model 1: adjusted for age and energy.

Model 2: additionally, adjusted for region, marital status, education, disease history, physical activity, family history of breast cancer, menopausal status, smoking, alcohol consumption, and socioeconomic status.

Model 3: additional adjustment for BMI.

ORs were obtained from binary logistic regression.

## Discussion

In the present study, we found a significant inverse relationship between the Mediterranean diet and the odds of breast cancer. After controlling for potential confounders, this association remained significant. Among postmenopausal women, a significant inverse association was found, while among premenopausal women, this association was not significant. To the best of our knowledge, this is the first study on the Middle Eastern population that examined the relationship between the Mediterranean dietary pattern and breast cancer.

Breast cancer is one of the most common cancers among women (24, 25). Previous studies on diet–breast cancer relationships revealed that diet as a modifiable risk factor has an important contribution to the etiology of cancers such as breast cancer (24–27). Recent studies have mainly investigated the relationship between individual food groups or nutrients with breast cancer, and the influence of a whole diet has less been studied (28). In the current study, an inverse association was found between the Mediterranean dietary pattern and the odds of breast cancer. Our findings were aligned with the findings from a case–control study conducted by Turati et al. in Switzerland in which adherence to the Mediterranean diet was inversely associated with the risk of breast cancer (15). The findings from a prospective cohort study conducted by Brandt et al. showed an inverse association between adherence to the Mediterranean dietary pattern and receptor-negative breast cancer (29). However, in the Swedish Women's Lifestyle and Health cohort study that was performed on 49,258 women aged 30–49 years, Couto et al. showed that the Mediterranean dietary pattern did not decrease breast cancer risk (16). Different findings of Couto et al. study might be due to the different age range participants compared with previous studies and also ours. Couto et al. recruited young women (30–49 years) and all of them are probably premenopausal women. It seems that the association between the Mediterranean diet and breast cancer is different between pre- and postmenopausal women; such that evidence of the inverse association among postmenopausal women is more than premenopausal ones. Moreover, in the current study, we found a significant inverse relationship between the Mediterranean diet and breast cancer among postmenopausal women but not among premenopausal women. In addition, different adjustments for confounding variables are another reason for the observed controversy.

The observed disparity between premenopausal and postmenopausal women might be explained by internal estrogen in premenopausal women. Internal estrogen can increase breast cancer risk by increasing reactive oxygen species (ROS) production in the breast (30, 31). In addition to causing genotoxicity through an indirect increase in genomic instability, excess ROS can stimulate breast carcinogenesis by triggering a redox–associated signaling pathway (32). Overall, the non-significant relationship between the Mediterranean dietary pattern and breast cancer in premenopausal women might be due to the interference of endogenous estrogenic effects with the beneficial effects of the Mediterranean diet.

Although the exact mechanisms through which the Mediterranean diet contributes to the prevention of breast cancer are unclear, this protective effect might be due to its nutrient content. Mediterranean diet contains a high amount of MUFAs and omega-3 fatty acids that have anti-inflammatory properties (21, 33). Inflammation is an important risk factor in the incidence of breast cancer (34). Mediterranean diet is rich in dietary fiber, lignans, flavonoids, and other compounds that are known to support estrogen metabolic pathways and have estrogen-modulatory effects which are protective against breast cancer among premenopausal and postmenopausal women (35–37). Antioxidant-rich foods such as fruits and vegetables are other components of the Mediterranean diet that can have protective effects against breast cancer by reducing the production of oxidant species (38).

This study has several strengths. First, we investigated the relationship between adherence to the Mediterranean dietary pattern and breast cancer in the Middle East. In addition, we controlled our analyses for many confounding variables. We presented a subgroup analysis based on menopausal status which is an important confounder in diet–cancer relationships. Furthermore, to collect data, we used validated questionnaires, which can support the accuracy of our findings. However, some limitations should be taken into account. Since selection and recall bias are common in case–control studies, our findings are subjected to these two types of bias. In addition, due to the use of FFQ, misclassification of study participants is inevitable. In this study, although we controlled our analyses for several confounding variables, we cannot exclude the potential effects of remaining confounding variables. In addition, since the dietary intakes of the Middle East nations are different from the Western population, caution should be taken in generalizing these findings to other populations.

## Conclusion

In conclusion, we found that adherence to a Mediterranean-style diet was associated with reduced odds of breast cancer in the Middle Eastern population. Such finding was also seen among postmenopausal women but not among premenopausal women. To confirm these findings, more studies should be conducted in diverse populations. In addition, future studies should determine this association for receptor-negative and receptor-positive breast cancer patients separately.

## Data availability statement

The raw data supporting the conclusions of this article will be made available by the authors, without undue reservation.

## Ethics statement

The studies involving human participants were reviewed and approved by Ethical Committee of the Isfahan University of Medical Sciences, Isfahan, Iran. The patients/participants provided their written informed consent to participate in this study.

## Author contributions

AE, SB-K, and LA contributed to the conception, design, and data collection. SB-K contributed to data preparation. OS, NE, and AE contributed to data analysis and manuscript drafting. AE supervised the study. All authors read and approved the final version of the manuscript.
